# Perovskite‐Graphene Heterostructure Biosensor Integrated with Biotunable Nanoplasmonic Ternary Logic Gate for Ultrasensitive Cytokine Detection

**DOI:** 10.1002/advs.202503124

**Published:** 2025-05-21

**Authors:** Jiaxing Sun, Lin Zhou, Zening Li, Guolin He, Hongju Mao, Jianlong Zhao, John A. Hunt, Xianfeng Chen

**Affiliations:** ^1^ School of Science and Technology Nottingham Trent University Nottingham NG11 8NS UK; ^2^ Key Laboratory of Transducer Technology Shanghai Institute of Microsystem and Information Technology Chinese Academy of Sciences Shanghai 200050 China; ^3^ General Surgery Center Department of Hepatobiliary Surgery II Zhujiang Hospital Southern Medical University Guangzhou Guangdong 510280 China; ^4^ Medical Technologies Innovation Facility Nottingham Trent University Nottingham NG11 8NS UK

**Keywords:** 2D materials, biosensor, cytokine, FET, graphene, heterostructure, LSPR, perovskite, ternary logic gate

## Abstract

The integration of 2D‐materials and optoelectronic devices has attracted great attention for advanced applications. We propose the first perovskite/graphene heterostructure‐based FET biosensor with uniquely biotunable ternary logic gating functionality. The biosensor integrates a lateral perovskite‐on‐graphene heterostructure phototransistor with a vertical bio‐nano‐photonic filter, with a decoupled construction inset. In the phototransistor, photoactive perovskite quantum dots (PQDs) serve as sensitizers to absorb light while a high mobility single‐layer graphene (SLG) acts as an expressway for carrier transport. In the bio‐nano‐photonic filter, a localized surface plasmon resonance (LSPR) is induced by gold nanoparticles (AuNPs) in conjunction with antigen‐antibody binding, tuning the delivery of light passing through the filter and facilitating biotunable functionality with ternary modes. The biosensor is set up to detect human interleukin‐6 (IL6) in order to determine and achieve ultrahigh sensitivity with a limit of detection (LOD) of 0.9 fg mL^−1^ (43 aM), which is 4 orders of magnitude greater than graphene‐FET biosensors. This ultrahigh sensitivity is achieved due to the synergistic effect of PQDs/SLG heterostructure, exhibiting superior electrical, optical, and physicochemical properties, consequently providing significantly high performance of the biosensor in terms of label‐free, ultrahigh sensitivity (attomolar level), rapid responsivity (5 min), excellent stability, and selectivity. This heterostructure‐based biotunable configuration could open a new avenue for 2D materials in the realm of next‐generation bio‐nano‐photonic platforms for applications in healthcare, early diagnosis, and rapid detection.

## Graphical abstract

This work proposes the first perovskite/graphene heterostructure‐based FET biosensor with uniquely biotunable ternary logic gating functionality for the detection of human IL6 cytokines. The biosensor integrates a lateral perovskite‐on‐graphene heterostructure phototransistor with a vertical bio‐nano‐photonic filter, achieving ultrahigh sensitivity with an extremely low LOD of 0.9 fg mL^−1^ (43 aM) and a broad detection range of 10^0^–10^5^ fg mL^−1^.

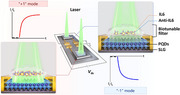

## 1 Introduction

Cytokines are crucial cell mediators in immune and inflammatory responses.^[^ ^1^ ^]^ The determining cytokine levels in a variety of biological fluids (i.e., serum, blood, and saliva) provide valuable information in relation to the diagnosis and prognosis of different diseases and trauma.^[^ ^2^, ^3^ ^]^ Extremely high cytokine secretion may result in organ failure and death. For example, patients with Sars Cov2 (COVID‐19) might suffer cytokine storm syndrome.^[^ ^4^, ^5^ ^]^ As one of the key human inflammatory cytokines, interleukin 6 (IL6) has an extensive impact on cells of the immune system and demonstrates hormone‐like traits that influence the homeostatic balance.^[^ ^6^ ^]^ The elevated circulating IL6 concentrations are concerned with numerous diseases, including cardiovascular disease,^[^ ^7^, ^8^ ^]^ cancer,^[^ ^9^ ^]^ type 2 diabetes,^[^ ^3^ ^]^ and severe acute COVID‐19 infection.^[^ ^10^, ^11^ ^]^ In general, it is required to detect IL6 concentrations down to pg mL^−1^ in human derived biological fluids.^[^ ^2^, ^12^ ^]^ Hence, it is crucial to develop biosensors with a limit of detection (LOD) in the range of sub pg mL^−1^. Enzyme‐linked immunosorbent assay (ELISA) has been the gold standard method for cytokine detection for many decades.^[^ ^13^ ^]^ However, these methods are laborious and costly, limiting their wide usage in applications, particularly in emergency and urgent point‐of‐care settings where the rapid screening and diagnosis can be lifesaving.

Among various sensing methods, 2D materials‐based field‐effect transistors (FETs)^[^ ^14^, ^15^, ^16^ ^]^ have attracted great research attention due to the advantages of label‐free detection, high sensitivity, and fast response.^[^ ^17^, ^18^, ^19^, ^20^, ^21^, ^22^, ^23^, ^24^, ^25^, ^26^, ^27^, ^28^, ^29^, ^30^, ^31^ ^]^ Hao et al. proposed graphene‐based FETs for IL6 detection with an LOD of hundreds of fM.^[^ ^17^, ^18^ ^]^ The molybdenum disulfide (MoS_2_)‐based FET biosensors were developed for cytokine detection with an LOD down to tens of fM.^[^ ^21^, ^22^, ^23^ ^]^ Very recently, van de Waals (vdW) heterostructures composed of atomically thin 2D materials have been extensively investigated, such as tin disulfide (SnS_2_)/hexagonal boron nitride (h‐BN), black arsenic (b‐As)/SnS_2_, and carbon nanomembrane (CNM)/graphene heterostructure‐based FET biosensors.^[^ ^29^, ^30^, ^31^ ^]^ The operating mechanism of the FET biosensor is to detect the electronic response induced by the binding between biomolecules. However, the electric field of FET is normally distributed throughout a bio‐sample solution, which could perturb signal stability or cause unwanted leakage current and/or short circuits. Another challenge of 2D materials‐based FETs is the degradation of 2D materials,^[^ ^14^, ^32^ ^]^ which will affect the stability over long‐term exposure to aqueous environments. An alternative scheme has been proposed to avoid the degradation by separating the FET detector and the biological solutions.^[^ ^23^ ^]^ However, the rapid and highly sensitive bio‐detection still remained as a major challenge.

The emerging perovskites exhibit excellent intrinsic optoelectronic properties, such as a direct bandgap, large absorption coefficient, and low manufacturing costs, making them promising candidates for numerous applications.^[^ ^33^, ^34^, ^35^ ^]^ The photo‐response can be enhanced by hybridizing perovskite with graphene, which is attributed to the efficient charge transfer from perovskite to graphene.^[^ ^36^, ^37^, ^38^ ^]^ Over the last few years, the perovskite and graphene combinations have been extensively developed for various applications, but the majority has been applied to optoelectronic applications such as solar cells,^[^ ^35^ ^]^ photodetectors,^[^ ^36^, ^37^ ^]^ and phototransistors.^[^ ^38^, ^39^ ^]^

**Figure 1:**
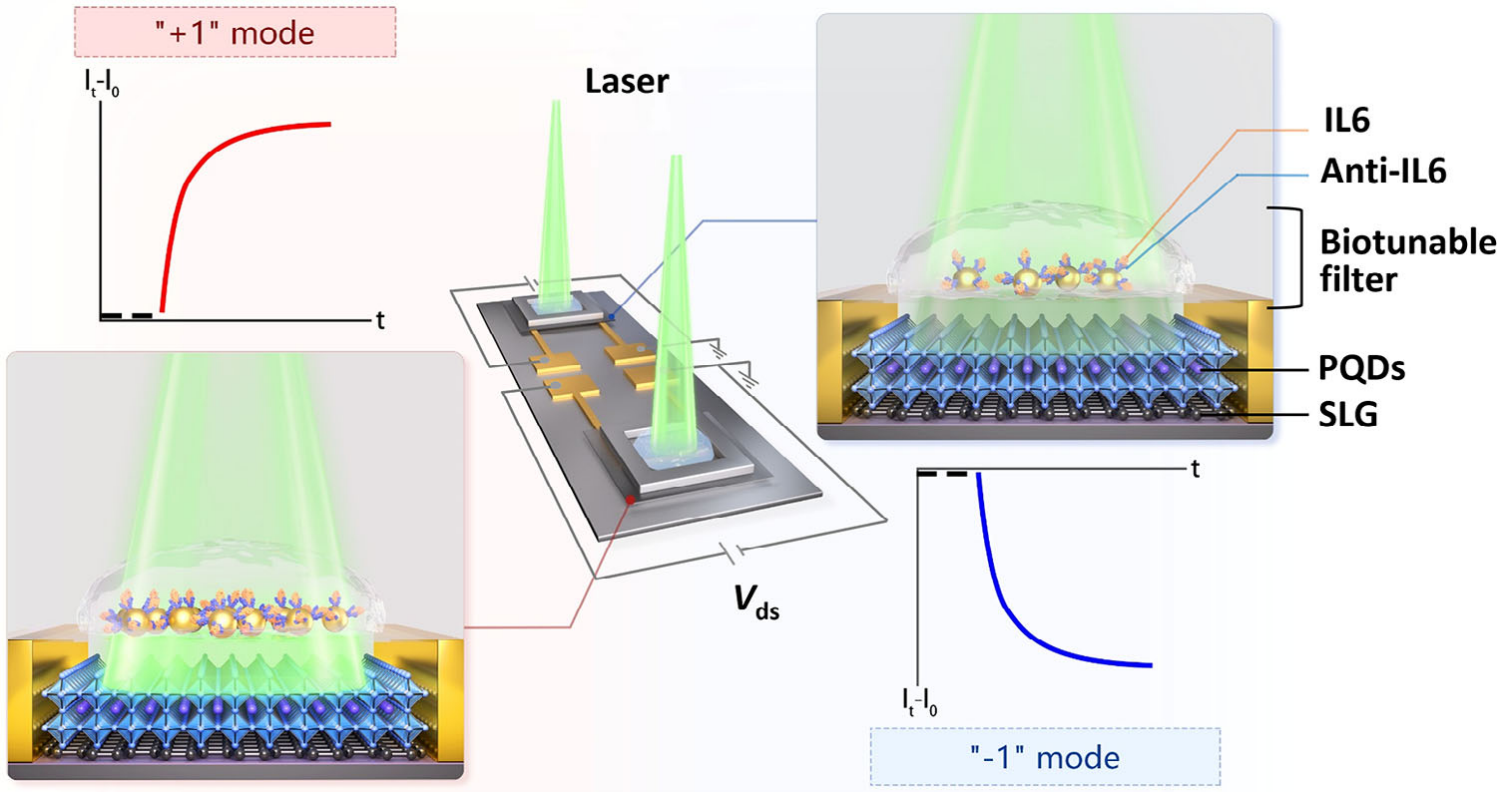
PQDs/SLG heterostructure FET biosensor with biotunable nanoplasmonic ternary logic gating functionality. The biosensor was integrated by a lateral perovskite‐on‐graphene heterostructure phototransistor and a vertical bio‐nano‐photonic filter consisting of anti‐IL6‐immobilized AuNPs. The biosensing principle is based on the LSPR shifts induced by antigen‐antibody binding, tuning the delivery of incident light passing through the bio‐nano‐photonic filter to the phototransistor.

Here, for the first time, we propose a perovskite/graphene heterostructure‐based FET biosensor integrated with a biotunable nanoplasmonic ternary logic gate for cytokine detection, operating with three modes (+1, 0, ‐1). As the diagram illustrated in **Figure** **1**, the biosensor is comprised of a lateral perovskite‐on‐graphene phototransistor integrated with a vertical bio‐nano‐photonic filter, with a decoupled construction inset. In the phototransistor, a thin film of perovskite quantum dots (PQDs) is spin‐coated upon a single‐layer graphene (SLG), where the photoactive PQDs capture the light and generate electro‐hole pairs, while the photogenerated charges are transferred to the high mobility graphene layer. The vertical bio‐nano‐photonic filter consists of an anti‐human interleukin 6 antibody (anti‐IL6) immobilized gold nanoparticles (AuNPs) on a SiO_2_ thin layer. Under laser illumination, the localized surface plasmon resonance (LSPR) induced by AuNPs in conjunction with the binding of antigen‐antibody tunes the delivery of incident light passing through the filter and reaching the underlying FET. In this work, we developed two types of filters (with high‐density AuNPs and low‐density AuNPs) to facilitate biotunable gating functionality with ternary logic modes (+1, 0, ‐1). The PQDs/SLG biosensor has been implemented for label‐free detection of human IL6 cytokines, demonstrating an ultrahigh sensitivity with an LOD as low as 0.9 fg mL^−1^ (43 aM) and ultra‐fast (5 min) detection. The two different 2D materials (PQDs and SLG) demonstrated a synergistic effect, with each enhancing the other's efficiency for light‐carrier transition and high‐speed electron transportation, consequently improving the sensor performance in terms of sensitivity, efficiency, and reliability. These results reveal that the heterostructure 2D materials‐based biosensor can play a key role in future applications.

## 2 Results and Discussion

### 2.1 Design and Mechanism of PQDs/SLG Heterostructure FET Biosensor

The biosensor was integrated by a lateral phototransistor and a vertical bio‐nano‐photonic filter (Figure 1). The FET device was fabricated by a combination of the metal lift‐off technique, graphene wet transfer method, and PQDs spin‐coating technology. The bio‐nano‐photonic filter was built by self‐assembly of AuNPs on a thin SiO_2_ layer, which was placed on the top of the PQDs film with a 100 µm physical gap. An incubation chamber was added on top of the filter to hold the biofluids containing IL6. A laser (λ = 532 nm) was used to launch the light vertically down to the filter and FET device. The detailed fabrication processes were described in the Experimental Section and Figure S1 (Supporting Information). In the lateral phototransistor, a perovskite‐on‐graphene heterostructure film bridged the FET source and drain electrodes, photoactive PQDs served as sensitizers to absorb light and convert the photons to electric carriers, while high mobility SLG acted as an expressway for carrier transport. With this architecture, PQDs/SLG demonstrated synergistic effects, with each enhancing the other's capabilities for efficient light‐carrier transition and high‐speed electron transportation. From the top of the device, the bio‐nano‐photonic filter tuned the delivery of incident light by means of LSPR shift induced by antigen‐antibody binding, operating as a biotunable gate with three logic modes (+1, 0, ‐1).^[^ ^40^ ^]^

**Figure 2:**
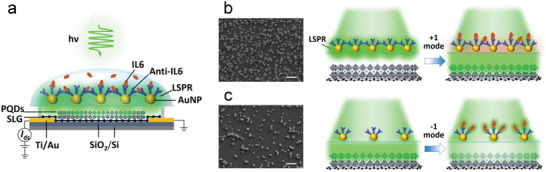
a) Schematic of biosensor with a decoupled construction between filter and phototransistor. The mechanism of the biotunable ternary logic gate is based on tuning the delivery of incident light to PQDs/SLG phototransistor by LSPR shifts induced by biomolecular binding by the use of: b) high‐density AuNPs filter for operating from 0 to +1 mode, c) low‐density AuNPs filter for operating from 0 to ‐1 mode (SEM image scale bar: 500 nm).

**Figure** **2** illustrates an insight into the mechanisms underpinning the biotunable ternary logic biosensor. As the cross‐section in Figure 2a, a decoupled construction (100 µm physical gap) was designed between AuNPs‐filter and PQDs film to separate the ‘wet’ biosensing area and the ‘dry’ photodetection area, which not only simplified the complexities inherent in traditional FETs but also enhanced device stability without impairing the properties of the 2D materials. Two types of filters were developed: high‐ and low‐ density AuNPs filters. SEM images show the high‐density filter (Figure 2b) demonstrated high‐uniformity in the distribution of AuNPs upon the SiO_2_ layer with a high‐density of ≈50.6 particles µm^−2,^ while the counterpart (Figure 2c) showed less uniformity in features with a low‐density of ≈20.8 particles µm^−2^.

For the high‐density filter (Figure 2b) under laser illumination (0 mode), the initial photocurrent detected by the phototransistor was lower due to the strong LSPRs excited by the high‐density and well‐distributed gold nanoparticles under laser illumination, blocking the incident light passing through the filter. When the target human IL6 cytokines were present to bind with the anti‐IL6 probe, a red shifted LSPR was induced due to the change of the local refractive index near the AuNPs surfaces,^[^ ^23^, ^27^ ^]^ hence a larger amount of incident photons was transmitted through the filter increasing the photocurrent detected by the underlying phototransistor (+1 mode). The tuning was dependent on IL6 concentration as well as the antigen‐antibody binding time. For the low‐density filter (Figure 2c), a strong photocurrent was initially detected (0 mode) due to very limited LSPRs generated by the poor‐distributed low‐density AuNPs, where a large fraction of incident light passed through the filter. When affinity binding increased, more antigens were attached to antibodies, shielding the light transportation and hence the photocurrent decreased (‐1 mode).

### 2.2 2D Materials Surface Morphological Characterization

**Figure 3:**
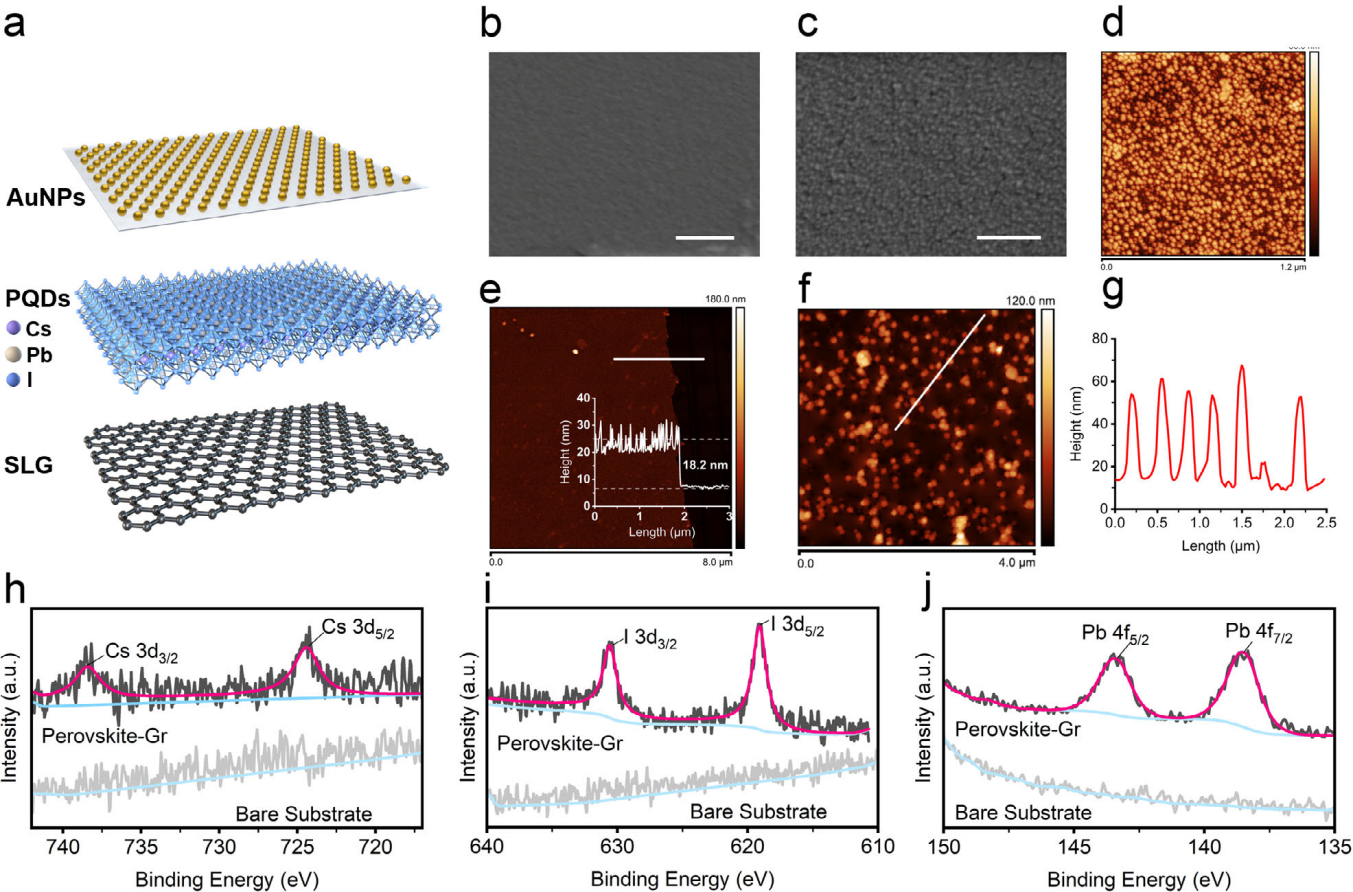
a) Schematic of the PQDs/SLG heterostructure and AuNPs filter. SEM images of b) SLG covered on FET substrate, c) PQDs thin film spin‐coated upon graphene (scale bar: 500 nm). AFM images of d) PQDs film surface, e) PQDs/SLG heterostructure with height profile (inset), f) high‐density AuNPs filter, g) AuNPs height profile graph extracted from f). High‐resolution XPS spectra of CsPbI_3_ perovskite/graphene heterostructure for (h) Cs, (i) I, and (j) Pb elements.

The schematic of PQDs/SLG heterostructure and AuNPs‐assembled filter is illustrated in **Figure** **3**a. The surface morphologies were characterized by atomic force microscopy (AFM), scanning electron microscope (SEM), and X‐ray photoelectron spectroscopy (XPS).

For the designed FET, the drain and source Ti/Au electrodes with a height of 57.1 nm were fabricated on a SiO_2_/Si substrate with a channel length of 2.5 µm (Figure S2, Supporting Information). A single‐layer graphene was transferred to cover the FET channel and electrodes firmly and smoothly (Figure 3b), providing good contact. Then, perovskite quantum dots (CsPbI_3_) were spin‐coated upon SLG to form a granular thin film (Figure 3c), which generated a rougher surface than graphene.^[^ ^41^ ^]^ The surface morphology of the heterostructure was characterized by AFM (Figure 3d) and demonstrated an increased surface roughness with a root‐mean‐square (RMS) roughness of 4.8 nm. The thickness of the PQDs/SLG heterostructure layer was measured by the height profile of the boundary, showing a thickness of 18.2 nm (Figure 3e). The high‐density AuNPs filter (Figure 3f) showed a uniform distribution of AuNPs. By reviewing a typical profile line across the filter surface (Figure 3g), there were 8 AuNPs well‐distributed with an average spacing of 280 ± 5 nm and a height of 50 ± 10 nm. The surface morphology of the low‐density filter was also examined (Figure S3, Supporting Information), demonstrating an irregular distribution of AuNPs over the filter surface. The XPS measurements (Figure S4, Supporting Information) were used to analyze the perovskite elements. The XPS spectra of CsPbI_3_ exhibited i) two typical characteristic peaks at 724.4 and 738.4 eV that corresponded to the elements of Cs 3d_5/2_ and Cd 3d_3/2_, respectively (Figure 3h); ii) two characteristic peaks at 619.1 and 630.6 eV that corresponded to the elements of I 3d_5/2_ and I 3d_3/2_, respectively (Figure 3i); and iii) two characteristic peaks at 138.6 and 143.4 eV which corresponded to the elements of Pb 4f_7/2_ and Pb 4f_5/2_, respectively (Figure 3j).^[^ ^41^, ^42^ ^]^

### 2.3 Optical Characterization of PQDs/SLG Heterostructure FET

**Figure 4:**
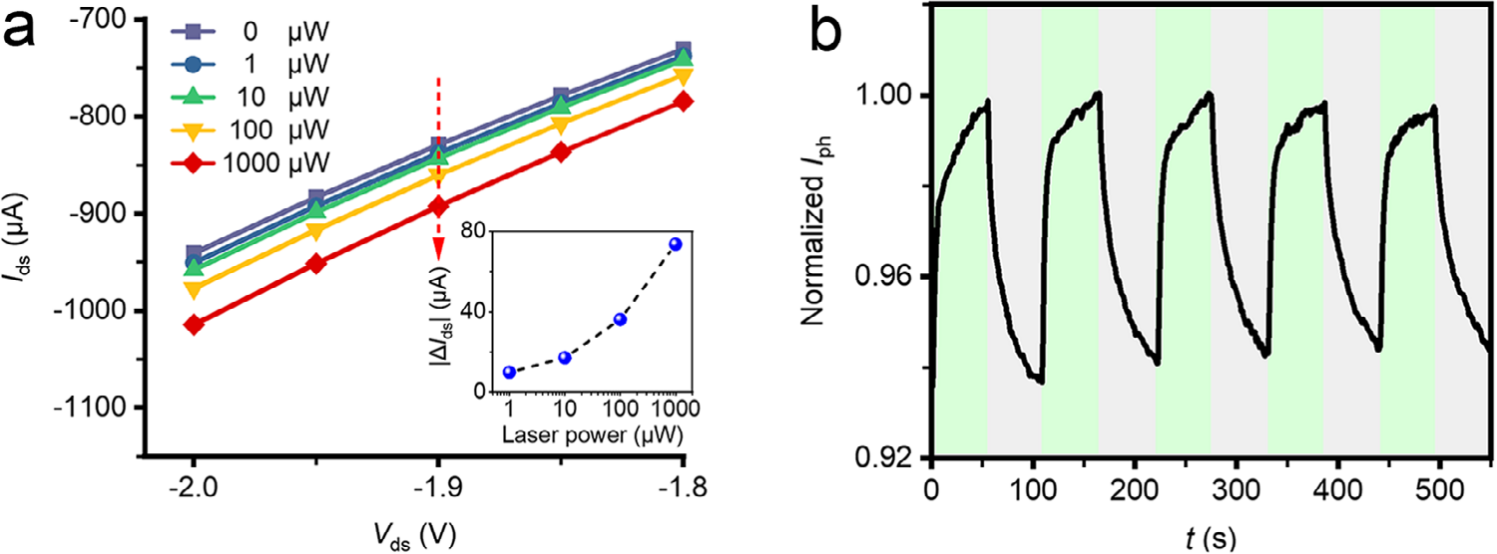
a) *I_ds_*‐*V_ds_* curves of perovskite/graphene heterostructure FET under laser illumination with different powers (inset: the dependence of photocurrent against laser power at *V_ds_* = −2 V). b) Temporal *I_ph_* response under alternating dark and light (*λ* = 532 nm, *P* = 500 µW).

The effect of the drain bias applied to the FET was plotted in **Figure** **4**a. The photocurrent *I*_ds_ was recorded while the source‐drain voltage *V*_ds_ was swept from −2.0 to −1.8 V under the laser illumination with varying power (0, 1, 10, 100, and 1000 µW), revealing a noticeable increase in *I*_ds_ against the increased laser power. As an example (with a laser power of 1 µW), the photocurrent increased from −950.7 to −737.6 µA when the applied bias voltage was changed from −2.0 to −1.8 V. As a function of the incident laser power, for a fixed *V*_ds_ = −2.0 V, as shown in the inset figure in Figure 4a, the photocurrent *I*_ds_ changed 63.7 µA when the laser power increased from 1 to 1000 µW. Figure 4b shows the photo‐switching response of the proposed device. The normalized photocurrent *I*_ph_ was defined by the ratio of photocurrent *I*_ds_ over the maximum drain‐source current *I*_ds_max_ when the bias voltage was set as 0.1 V. The normalized photocurrent was stable as a function of time under the alternating illuminated and dark conditions with a duration of 55 s and a laser power of 500 µW. The On/Off photo‐responses were well maintained for five repeated cycles. The high photoresponsivity of the proposed device facilitated its capability for rapid and ultrasensitive biosensing applications.

### 2.4 Ultrasensitive Detection of IL6 Cytokines

The proposed device was able to detect IL6 cytokines. The anti‐human IL6 antibody‐immobilized AuNPs filter was loaded with human IL6 cytokine of different concentrations. During the antigen‐antibody binding events, the corresponding photocurrent *I*_ds_ was monitored in real‐time. The standard curve of photocurrent‐time response provided the best Hill model fitting for the experimental data, where the photocurrent enhancement Δ*I* can be described by the Hill‐Langmuir equation:^[^ ^43^, ^44^ ^]^

(1) $$\begin{array}{c} \Delta I=\frac{C_{IL6}^{n}}{C_{IL6}^{n}+K_{d}^{n}}\Delta I_{max} \end{array}$$

where *C_IL6_* is the ligand concentration, *n* is the Hill coefficient, which can determine the cooperative degree of ligand binding, *K_d_* is the dissociation constant of ligand molecular interaction, and ∆*I*_max_ is the maximum photocurrent enhancement.

#### 2.4.1 Under “0” Mode

For “0” mode (i.e., the AuNPs‐filter was immersed in 1×PBS buffer only), the base photocurrents *I*_ds_ of the biosensor were measured and plotted in Figure S5 (Supporting Information). The *I*_ds_ ‐ *V*_ds_ curves showed a non‐linear relationship: when a bias voltage increased to 1 V, the photocurrent *I*_ds_ was 256.04 µA and 267.46 µA for the high‐ and low‐ density filters, respectively. As discussed in Section 2.1, high‐density AuNPs excited stronger LSPR yielding less light flux to the phototransistor, hence its base *I*_ds_ was lower than that of the low‐density filter.

#### 2.4.2 Detection of IL6 Cytokines Under “+1” Mode

**Figure 5:**
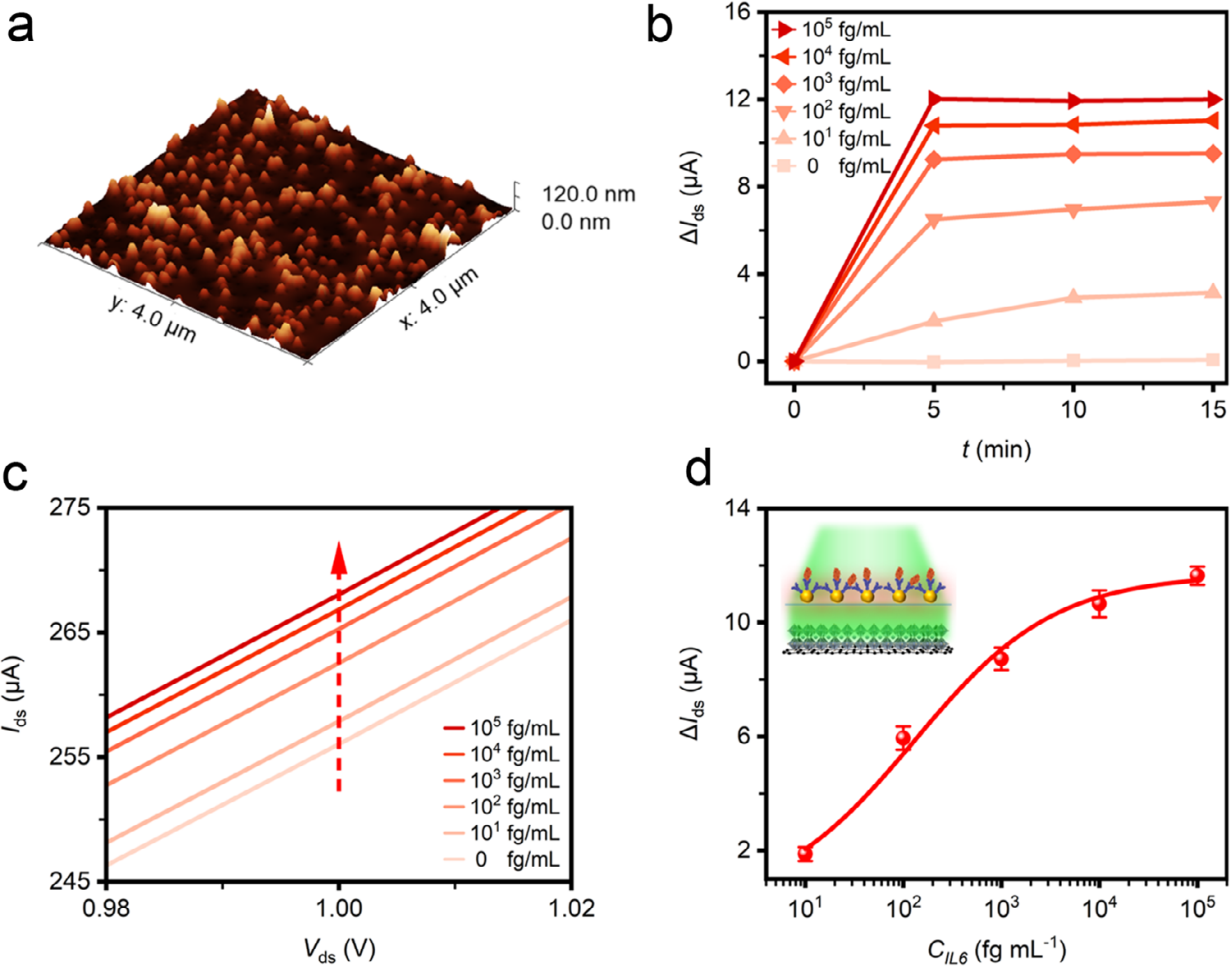
Ultrasensitive bio‐detection of IL6 cytokines under “+1” mode. a) 3D AFM image of high‐density AuNPs filter. b) Photocurrent variation Δ*I*_ds_ *(t*_n_) (at a bias *V*_ds_ = 1.0 V) during binding incubation with different IL6 concentrations. Each curve was measured under the same laser illumination (*λ* = 532 nm, *P* = 500 µW). c) *I*_ds_‐*V*_ds_ curves for different IL6 concentrations (at an incubation time of 5 min). d) Standard curve of PQDs/SLG‐based biosensor incorporating biotunable ternary logic gate under “+1” mode.

For operating on “+1” mode, the AuNPs filter with high‐density (**Figure** **5**a) was employed. The *I*_ds_‐*V*_ds_ were monitored as a function of increasing incubation time (Figure S6a, Supporting Information), and the photocurrent variations *ΔI*_ds_ *(t_n_)* were measured (at *V*_ds_ = 1.0 V) for the IL6 concentrations ranging from 0 to 10^5^ fg mL^−1^ (results in Figure 5b). At the initial “0” mode, the high‐density and well‐distributed AuNPs induced extremely strong LSPR to block the light passing through, remaining at the lower initial current. By loading IL6 cytokine in solution to the filter, a red shift of the LSPR peak was induced due to the local refractive index change accompanying the antigen‐antibody binding,^[^ ^23^, ^27^ ^]^ yielding a larger fraction of incident photons passing through the filter and reaching the photoconductive device. As depicted in Figure 5b, in the first 5 min, there was a phase of rapid antibody‐antigen binding during which the photocurrent increased dramatically, then the reaction rate began to asymptote to a steady‐state and gradually achieved a stable‐state. The rapid reaction and detection response of 5 min was 100% faster than that of a single graphene‐FET biosensor.^[^ ^17^ ^]^ As shown by *I*_ds_‐*V*_ds_ curves (Figure 5c), the photocurrent increased as a function of increasing the IL6 concentration. As an example, at a bias voltage of 1.0 V and an incubation time of 5 min, the photocurrent increased from 256.0 to 267.9 µA *(ΔI*_ds_ = 11.9 µA) when IL6 concentration increased from 0 to 10^5^ fg mL^−1^. Based on the standard calibration curve of the biosensor (Figure 5d), the LOD was as low as 3.7 fg mL^−1^, showing an ultrahigh sensitivity for cytokine detection. The Hill equation offered a method to quantify the degree of interaction between antigen‐antibody binding. From Equation 1, the calculated value of ∆*I*_max_, *K_d_*, and *n* was 4.62, 87.92 fg mL^−1^, and 0.47, respectively. The Hill coefficient *n* was less than 1, indicating a negative cooperativity in ligand binding to the receptor. To verify the reliability and reproducibility, three devices with high‐density AuNP filters (under “+1” mode) were used to detect IL6 with different concentrations (Figure S7, Supporting Information).

#### 2.4.3 Detection of IL6 Cytokines Under “‐1” Mode

**Figure 6:**
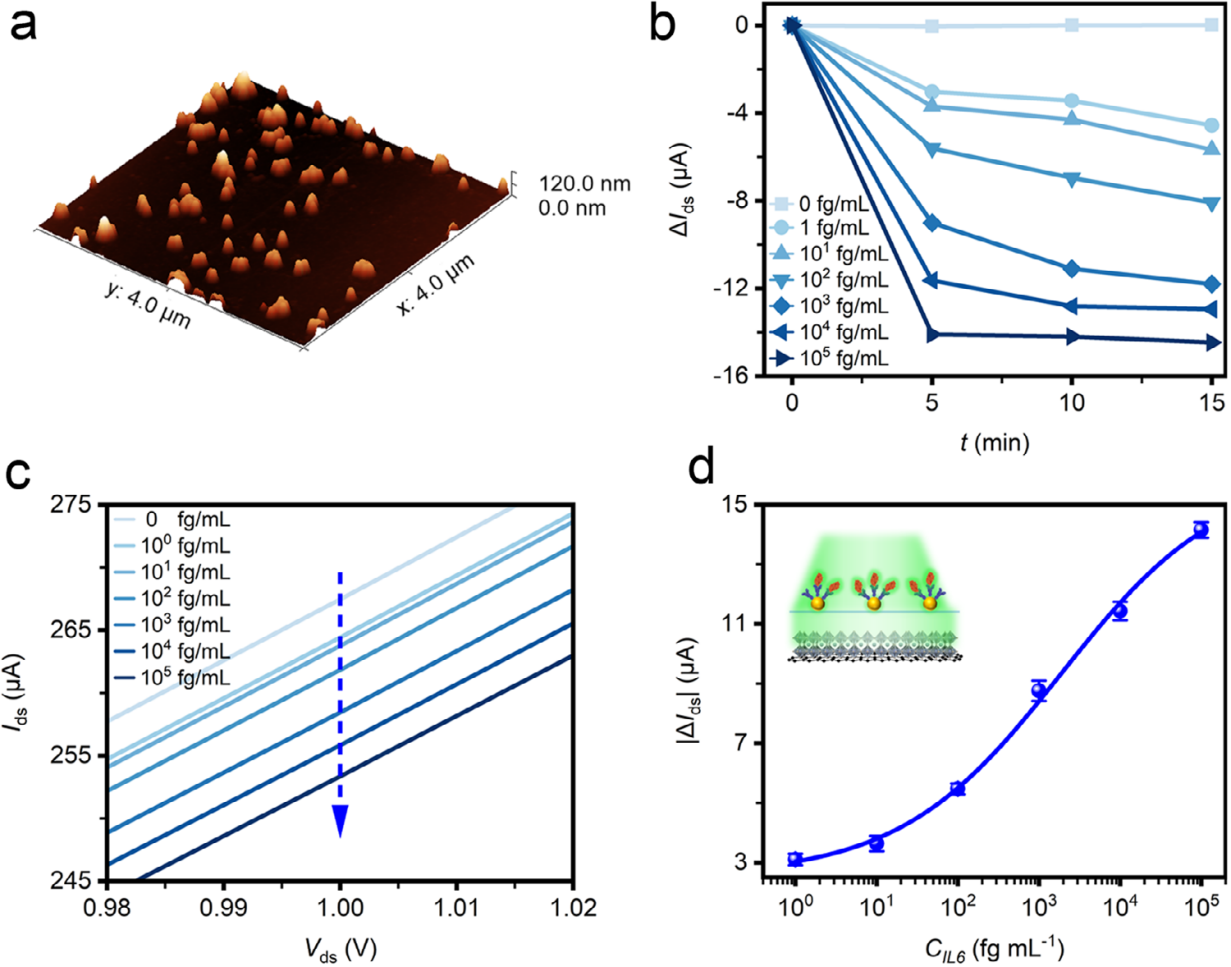
Ultrasensitive bio‐detection of IL6 cytokines under “‐1” mode. a) 3D AFM image of the low‐density AuNPs filter. b) Photocurrent variation Δ*I*_ds_ *(t*_n_) (at a bias *V*_ds_ = 1.0 V) during antigen‐antibody binding with different IL6 concentrations. c) *I*_ds_‐*V*_ds_ curves for different IL6 concentrations (at an incubation time of 5 min). d) Standard curve obtained with the biosensor under “‐1” mode.

While operating on “‐1” mode, the low‐density AuNPs filter (**Figure** **6**a) was used. The *I*_ds_‐*V*_ds_ response was monitored (Figure S6b, Supporting Information), and the photocurrent variations with increasing binding time were plotted in Figure 6b, which demonstrated the antigen‐antibody kinetic binding processes. At the initial “0” mode, a high photocurrent was measured as a large proportional of incident light was easily transmitted through the filter due to a lower LSPR induced by the limited amount of AuNPs. By loading IL6 solutions onto the anti‐IL6‐AuNPs filter, the accumulation of antigen‐antibody binding prevented the transmission of light through the filter. Consequently, the photocurrent decreased over the binding processes. As the *I*_ds_‐*V*_ds_ curves against IL6 concentrations shown in Figure 6c, the photocurrent decreased as the IL6 concentrations increased. At a bias voltage of 1.0 V and time of 5 min, the photocurrent decreased from 267.5 to 253.4 µA (Δ*I*_ds_ = −14.1 µA) when IL6 concentration increased from 0 to 10^5^ fg mL^−1^. Based on the standard calibration curve (Figure 6d), the PQDs/SLG biosensor demonstrated an extremely high sensitivity with an LOD as low as 0.9 fg mL^−1^ (43 aM), which was 4 orders of magnitude more sensitive than conventional graphene‐FET biosensors.^[^ ^17^, ^18^ ^]^ The enhanced performance could be attributed to the PQDs/SLG heterostructure, the ultrathin 2D‐heterostructured materials exhibited superior electrical, optical, and physicochemical properties than their pristine materials due to the synergistic characteristics of the two different 2D materials.^[^ ^45^ ^]^ The experimental data were fitted with the Hill model equation, and the calculated values of ∆*I*_max_, *K_d_*, and *n* were 13.57 µA, 1876.20 fg mL^−1^, and 0.44, respectively. The Hill coefficient *n* is less than 1, showing a negative cooperativity in ligand binding to the receptor. Similarly, three devices with low‐density AuNP filters (under “‐1” mode) were utilized for IL6 detection with results shown in Figure S8 (Supporting Information), demonstrating good reliability and reproducibility.

### 2.5 Specificity of the Biotunable Ternary Logic Biosensor

**Figure 7:**
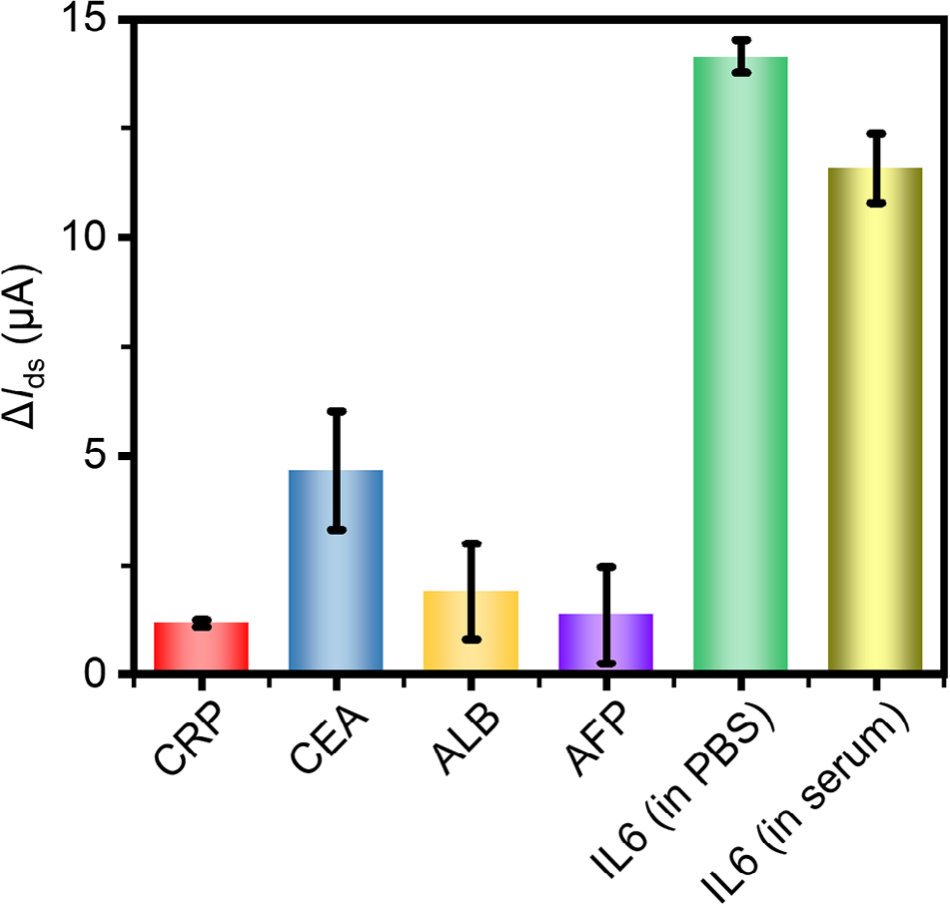
Specificity of the PQDs/SLG biosensor, showing high selectivity specific to IL6, as the signals for IL6 (in PBS and in serum) are distinctly higher than other non‐specific analytes.

It was well known that the avidin‐biotin complex is the established example of a strong noncovalent interaction between a protein and a ligand.^[^ ^46^, ^47^ ^]^ Compared to streptavidin, the neutral isoelectric point of neutravidin could minimize nonspecific binding caused by electrostatic interactions. Neutravidin provided a powerful and universal tool for biotin binding surfaces to construct different kinds of immunosensors. The specificity of the PQDs/SLG biosensor toward IL6 was evaluated to detect non‐specific analytes (all with the same concentration of 100 pg mL^−1^, and by using the low‐density filter). **Figure** **7** shows the results of the extended selectivity test by employing a variety of analytes, including CRP, CEA, ALB, and AFP. The maximum photocurrent variations were measured as 1.17, 4.67, 1.89, 1.36, and 14.15 µA for CRP, CEA, ALB, AFP, and IL6, respectively. The maximum signals for CRP, CEA, ALB, and AFP were only 8.2%, 33.0%, 13.4%, and 9.6% of IL6, respectively, indicating a high selectivity to IL6. To explore the interference in actual samples, the PQDs/SLG biosensor was evaluated toward serum samples and IL6 in serum (with IL6 concentration of 100 pg mL^−1^). The photocurrent variation induced by IL6 in serum was ≈82% of that observed in PBS.

## 3 Conclusion

We propose the first perovskite/graphene heterostructure‐based FET biosensor with uniquely biotunable ternary logic gating functionality for label‐free, ultrasensitive, and rapid detection of human IL6 cytokines. PQDs/SLG heterostructure 2D materials were integrated into a FET, producing synergistic effects that enhanced the efficiency of light‐carrier transition and high‐speed electron transportation. An antibody‐conjugated AuNPs filter acted as a tunable gate (with three logic modes: +1, 0, ‐1), by tuning the delivery of incident light that passed through the filter due to the LSPR shift induced by antibody‐antigen binding. In particular, a decoupled construction was designed to separate the ‘wet’ biosensing area and the ‘dry’ photodetection area to enhance device stability without impairing the properties of the 2D materials. The proposed biosensor was proven to detect human IL6 cytokine, achieving ultrahigh sensitivity with an LOD as low as 0.9 fg mL^−1^ (43 aM) and a fast response time, with a sample‐to‐answer time of 5 min, which was 4 orders of magnitude more sensitive and 100% faster than graphene‐FET biosensors. In addition, the specificity was investigated by running the analysis using CRP, CEA, ALB, and AFP analytes. These results demonstrated excellent selectivity toward the IL6 cytokine. A detailed comparison of different 2D materials‐based FET biosensors is presented in **Table** **1**. The proposed PQDs/SLG biosensor in this work exhibited ultrahigh sensitivity, extremely low LOD (to attomolar level), rapid response (5 min), a broad concentration detection range (6 orders of magnitude: 10^0^–10^5^ fg mL^−1^), and good stability and selectivity. The proposed heterostructure 2D materials‐based biosensor provided a remarkable bio‐nano‐photonic platform for biosensing with the advantages of a label‐free approach, rapid detection, and ultrahigh sensitivity and selectivity.

**Table 1:** Performance comparison of 2D materials‐based FET biosensors for bio‐detection.

Sensor type	2D materials	Target analyte	LOD	Response time	Detection range	Refs.
**Single 2D material – based FET biosensors**						
FET	Graphene	IL6	139 fM	10 min	2 pM ‐ 100 nM	[17]
FET	Graphene	IL6	611 fM	7 min	0.01 ‐ 100 nM	[18]
FET	Graphene	Exosomes	0.1 µg/mL	30 min	1 ‐ 100 pM	[19]
Fiber optic FET	rGO	Thrombin	0.2 nM	15 min	1.6 ‐ 25 nM	[20]
FET	MoS_2_	Streptavidin	1 fM	23 min	1 ‐ 300 fM	[21]
FET	MoS_2_	TNF‐α	10 fM	–	10 fM ‐ 1 nM	[22]
Dual‐mode FET	MoS_2_	IL‐1β	250 fg/mL (14 fM)	10 min	10^−1^ ‐ 10^3^ pg/mL	[23]
Dual‐mode FET	MoS_2_	Opioid peptide	0.1 nM	–	0.1 ‐ 100 nM	[24]
FET	WSe_2_	SARS‐CoV‐2	25 fg/µL	–	25 fg/µL‐1 ng/µL	[25]
Impedimetric sensor	Black phosphorene	IL6	1 pg/mL	10 min	0.003‐75 ng/mL	[26]
**Hybrid 2D materials – based FET biosensors**						
FET	AuNPs on graphene	Hepatitis B	50 pg/mL (2.08 pM)	15 min	0.1 ‐ 1000 pg/mL	[27]
FET	AuNPs/Graphene	IL6	1.6 fM	10 ‐ 20 min	31 fM ‐ 31 nM	[28]
**Heterostructure 2D materials – based FET biosensors**						
FET	SnS_2_/h‐BN	Streptavidin	0.5 pM	13.2 s	0.5 ‐ 100 pM	[29]
FET	b‐As/SSnS_2_	Streptavidin	1 pM	12.5 s	1 ‐ 100 pM	[30]
FET	CNM/Graphene	IL8	1 pM	5 min	0.5 ‐ 500 pM	[31]
Ternary‐mode FET	PQDs/Graphene	IL6	0.9 fg/mL (43.0 aM)	5 min	1 ‐ 10^5^ fg/mL	This work^a)^

^a)^ indicating the significance of the results.

## 4 Experimental Section

### Materials and Reagents

Single‐layer graphene (SLG) fabricated by chemical vapor deposition on copper was acquired from 2D Carbon Ltd. (Changzhou, China). CsPbI_3_ perovskite quantum dots (PQDs) were purchased from Mesolight Ltd. (Suzhou, China). Gold nanoparticles (AuNPs, d = 50 nm) were purchased from JCnano Tech Ltd. (Nanjing, China). Acetone, Ethanol, and Ammonium persulfate ((NH_4_)_2_S_2_O_8_) were purchased from Shanghai Lingfeng Chemical Reagent Co. Ltd. (Shanghai, China). (3‐Aminopropyl)triethoxysilane (APTES), Albumin (ALB), and Alpha‐fetoprotein (AFP) were purchased from Sigma–Aldrich (Wuxi, China). Deionized water was produced by a Milli‐Q purification system (Massachusetts, USA). Goat Anti‐Mouse IgG ‐ AF488 and Serum were obtained from Signalway Antibody LLC and Shanghai Xinfan Biotechnology Ltd, respectively. Phosphate‐buffered saline (PBS), Neutravidin, and SuperBlock were purchased from Thermo Fisher Scientific Inc. (Massachusetts, USA). Mouse biotinylated anti‐human IL6 antibody (anti‐IL6) and Recombinant human IL6 protein were purchased from Beijing Bioss Ltd. (Beijing, China). C‐reactive protein (CRP) and Carcinoembryonic antigen (CEA) were obtained from Hytest Ltd. (Turku, Finland) and Fitzgerald Ltd. (Acton, USA), respectively.

### Fabrication of Perovskite‐On‐Graphene Heterostructure Based FET

The methods, including photolithography, metal sputtering, and lift‐off, were utilized to fabricate the FET device with Ti(5 nm)/Au(52 nm) electrodes as the drain and source contacts on SiO_2_/Si substrate (Figure S1a, Supporting Information). Single‐layer graphene (SLG) on Cu film was first cut into 5 × 5 mm pieces and immersed into 0.1 mg mL^−1^ (NH_4_)_2_S_2_O_8_ solution for 4 h to etch away Cu, then the graphene pieces with PMMA were transferred into DI water and followed by transferring onto SiO_2_ substrate to cover the drain/source electrodes and the FET channel. Subsequently, the graphene‐FET was stabilized by placing it in an oven at 80°C for 30 min, the PMMA was removed with acetone, followed by washing with ethanol and DI water, and drying thoroughly. Finally, the CsPbI_3_ perovskite quantum dots (PQDs) with a concentration of 2.5 µg mL^−1^ were spin‐coated (800 rpm, 10 s) onto a graphene layer and crystalized by soft annealing at 80 °C for 30 min under an N_2_ environment.

### Fabrication and Biofunctionalization of Nano‐Photonic Filter

Distribution of AuNPs on a transparent substrate was used for LSPR biosensors. As shown in Figure S1b (Supporting Information), a thin SiO_2_ substrate was rinsed with acetone, isopropanol, and DI water, then the surface was treated with O_2_ plasma for 2 min at a power of 150 W, and the SiO_2_ substrate was incubated in a 10% APTES solution for 2 h. AuNPs were drop‐casted on the upper‐surface of the substate by incubating the substrate in the AuNPs solution overnight with concentrations of 50 and 5 µg mL^−1^ to fabricate high‐ and low‐ density AuNPs filters, respectively. Subsequently, the AuNPs self‐assembled filter was immersed in NeutrAvidin solution (0.1 mg mL^−1^) at 4°C overnight. The 50 µL 0.1 mg mL^−1^ biotinylated anti‐human IL6 antibody (anti‐IL6) was loaded onto the filter at room temperature for 1 h to immobilize anti‐IL6 onto the AuNPs surfaces. The non‐bound anti‐IL6 antibodies were washed away with PBS buffer. The unreacted sites on AuNPs surfaces were passivated with SuperBlock to block the remaining active carboxylic groups and prevent non‐specific adsorption. Fluorescent characterization was employed to verify the modification efficiency of anti‐IL6 on the surface of the AuNPs filter (both for low‐ and high‐ density filters), as shown in Figure S9 (Supporting Information). Finally, the anti‐IL6 biofunctionalized AuNPs filter was assembled upon the PQDs/SLG, with a gap of 100 µm to form the sensor device.

### Measurement System

All electronic characteristics of the FET were monitored using a Keithley 4200 semiconductor parameter analyzer. The Coherent Compass 115M‐5 laser (*λ* = 532 nm) was used as a light source with intensity adjusted between 1 µW and 1000 µW using an optical attenuator (Thorlabs NDC‐50C‐4M). The surface morphology was examined by a Scanning Electron Microscope (SEM, JSM‐7100F LV, JEOL Ltd., Japan), Atomic Force Microscope (AFM, Bruker Dimension Icon, Bruker Ltd., USA), and X‐ray Photoelectron Spectroscopy (XPS, Thermo Fisher Scientific Inc., USA).

### Statistical Analysis

The OriginPro2023 was used for the pre‐processing and plotting of the experimental data. The height and roughness analysis of all AFM images was performed using Gwyddion 2.61. The standard curves shown in Figure 5d and Figure 6d (as well as in Figures S7 and S8, Supporting Information) were obtained with OriginPro2023 using the Hill model fit. For all the fittings, the coefficient of determination (R^2^) was greater than 0.98. The quantitative data (in Figures 5, 6, and 7; Figures S7b,d,f, and S8b,d,f, Supporting Information) were expressed as the means ± standard deviations (SDs), with each data point averaged from a minimum of three independent measurements.

## Conflict of Interest

The authors declare no conflict of interest.

## Author Contributions

J.S. and L.Z. contributed equally to this work. X.C., J.H., J.Z., H.M., L.Z., and J.S. conceived and planned the experiments. J.S. and L.Z. wrote the first draft of the manuscript. X.C. and J.H. revised the manuscript with comments from all other authors. J.S. and L.Z. performed the experiments. Z.L. assisted in the device fabrication. J.S. performed the characterization, including AFM and SEM. L.Z. performed the XPS measurement.

## Supporting information



Supporting Information

## Data Availability

The data that support the findings of this study are available from the corresponding author upon reasonable request.
