# Supplementary material for: Perovskite‐Graphene Heterostructure Biosensor Integrated with Biotunable Nanoplasmonic Ternary Logic Gate for Ultrasensitive Cytokine Detection
Source: Adv Sci (Weinh). 2025 May 21;12(29):e03124. doi: 10.1002/advs.202503124 (PMC12362746; doi:10.1002/advs.202503124)
Supplement: Supplementary file 1 — Supporting Information [file ADVS-12-e03124-s001.pdf]

ADVANCED SCIENCE
Open Access

Supporting Information

for *Adv. Sci.*, DOI 10.1002/advs.202503124

Perovskite-Graphene Heterostructure Biosensor Integrated with Biotunable Nanoplasmonic Ternary Logic Gate for Ultrasensitive Cytokine Detection

Jiaxing Sun, Lin Zhou, Zening Li, Guolin He, Hongju Mao*, Jianlong Zhao*, John A. Hunt and Xianfeng Chen**

Supporting Information

Perovskite-Graphene Heterostructure Biosensor Integrated with Biotunable Nanoplasmonic Ternary Logic Gate for Ultrasensitive Cytokine Detection

Jiaxing Sun, Lin Zhou, Zening Li, Guolin He, Hongju Mao*, Jianlong Zhao*, John A. Hunt, Xianfeng Chen**

J. Sun, X. Chen
School of Science and Technology, Nottingham Trent University, Nottingham NG11 8NS, United Kingdom
E-mail: xianfeng.chen@ntu.ac.uk

L. Zhou, Z. Li, H. Mao, J. Zhao
Key Laboratory of Transducer Technology, Shanghai Institute of Microsystem and Information Technology, Chinese Academy of Sciences, Shanghai 200050, China
E-mail: zhoulinzlw@mail.sim.ac.cn, hjmao@mail.sim.ac.cn, jlzhao@mail.sim.ac.cn

G. He
General Surgery Center, Department of Hepatobiliary Surgery II, Zhujiang Hospital
Southern Medical University, Guangzhou, Guangdong 510280, China

J. A. Hunt
Medical Technologies Innovation Facility, Nottingham Trent University, Nottingham NG11 8NS, United Kingdom

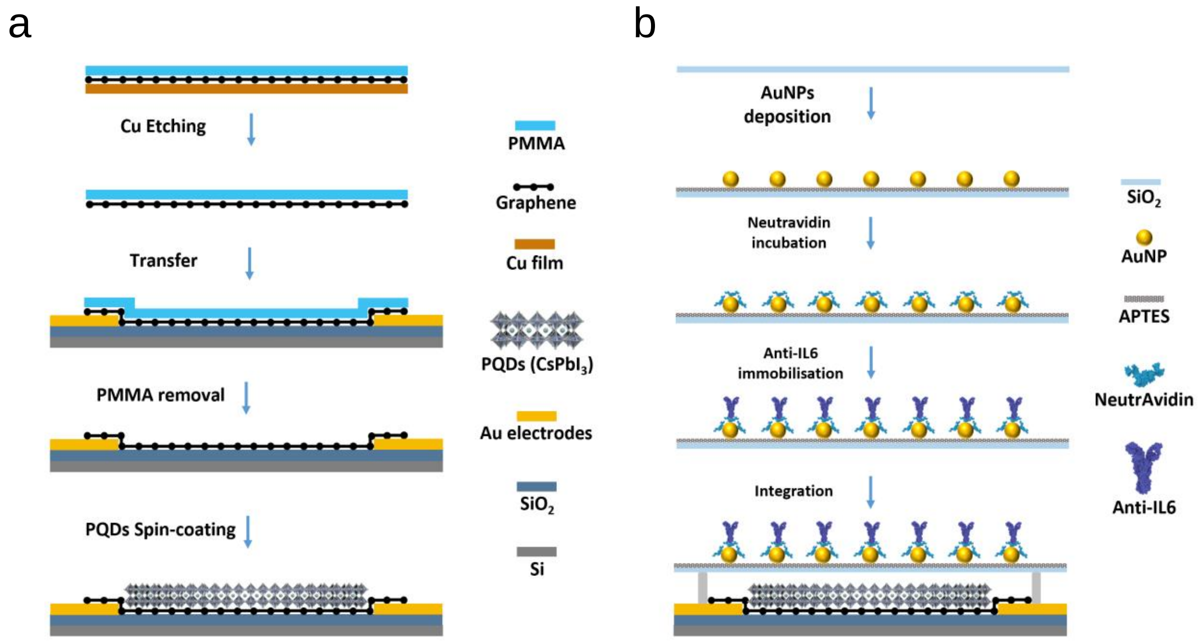

Figure S1. Schematic illustration of biosensor construction. a) Fabrication of perovskite-on-graphene heterostructure FET. b) Assembly of bio-nano-photonic filter.

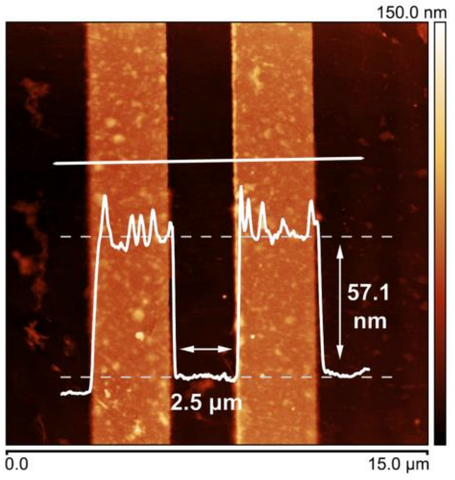

Figure S2. SEM image of perovskite-graphene heterostructure on FET drain/source electrodes with FET channel length of 2.5 µm (scale bar: 500 nm).

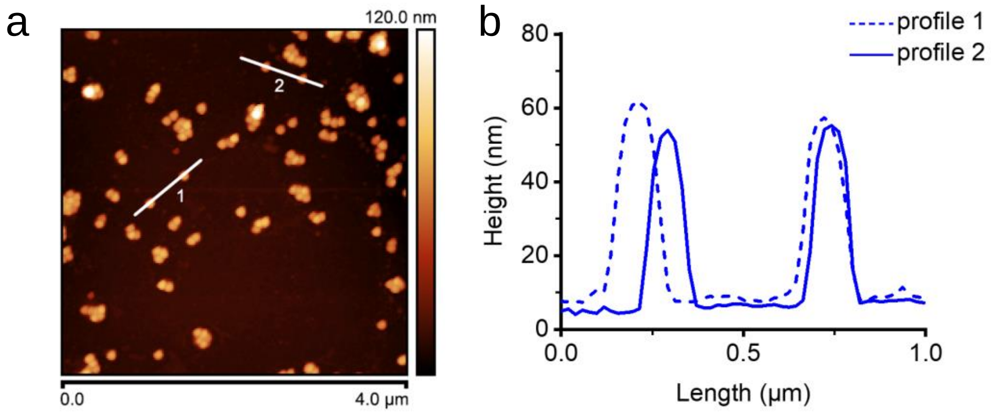

Figure S3. a) AFM image of low-density AuNPs filter. b) The corresponding height profiles of measured AuNPs in (a) along the white lines.

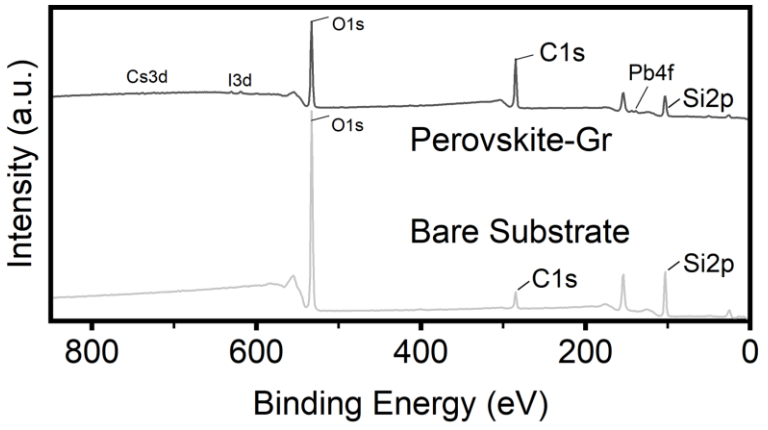

Figure S4. XPS spectra of PQDs ($CsPbI_3$)/graphene heterostructure on SiO_2/Si substrate.

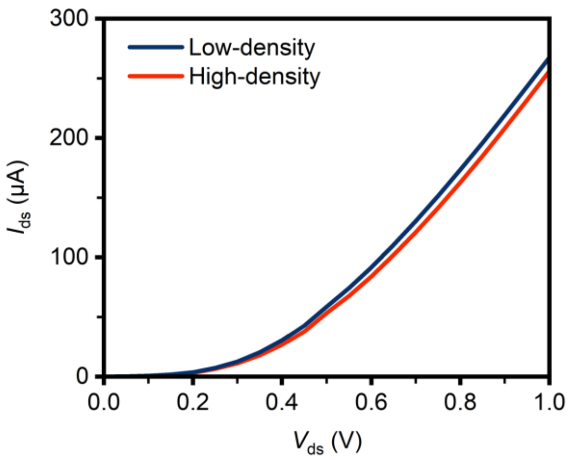

Figure S5. I_{ds}-V_{ds} curves for the baseline photocurrent of biosensor with high-density and low-density filters (λ = 532 nm, P = 500 μW).

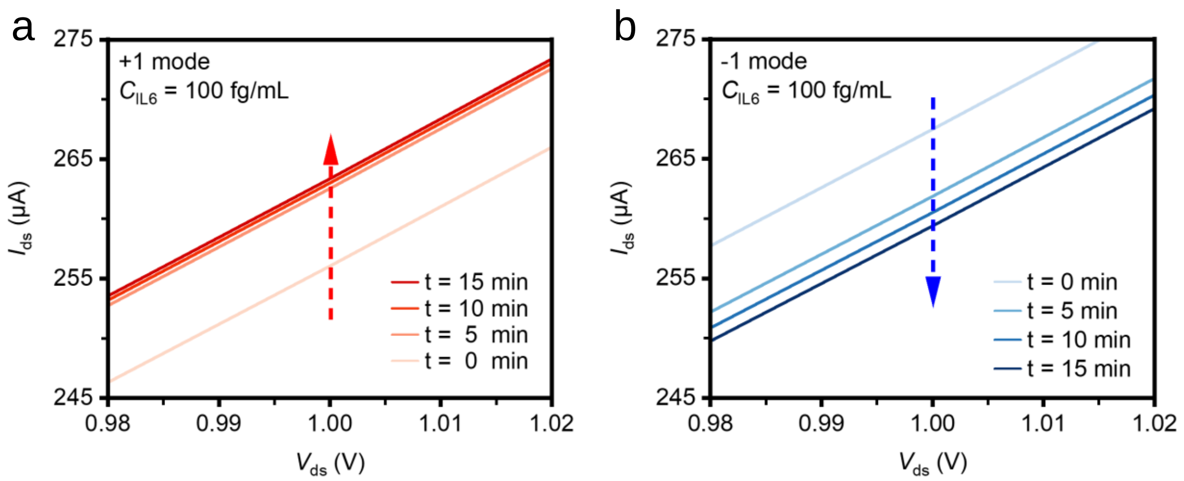

Figure S6. ΔI_{ds}-V_{ds} curves at different incubation time of 0, 5, 10, and 15 min with IL6 concentration of 100 fg mL^{-1} while the biosensor worked at a) "+1" mode and b) "-1" mode.

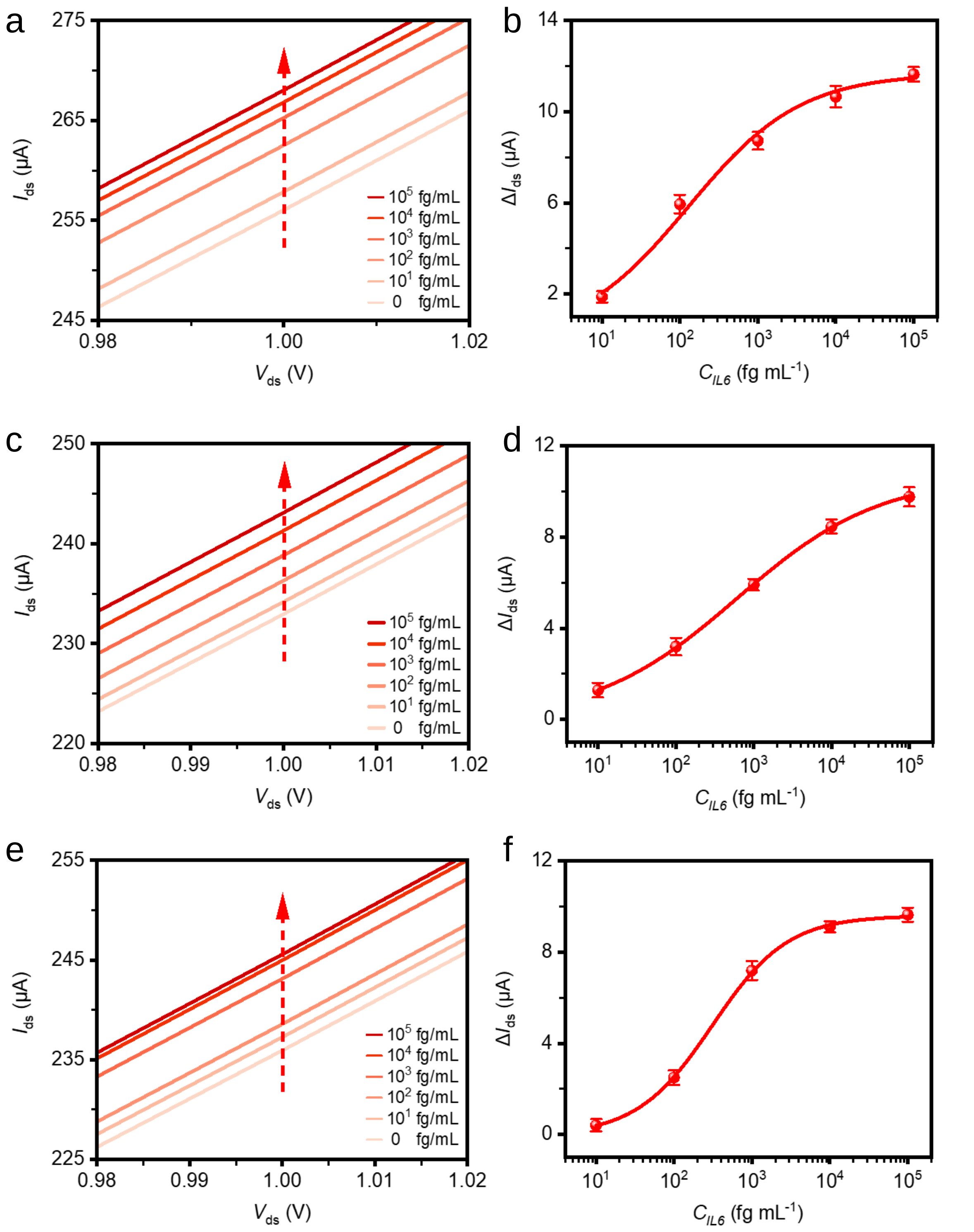

Figure S7. Detection of IL6 using Device-1, 3, 5 (under "+1" mode). The I_{ds}-V_{ds} curves for different IL6 concentrations (at incubation time of 5 min) of (a) Device-1 (shown as Figure 5c in paper), (c) Device-3, and (e) Device-5. The standard curves of (b) Device-1 (shown as Figure 5d in paper), (d) Device-3, and (f) Device-5 with ΔI_{ds} of 11.9 μA, 9.8 μA, and 9.6 μA, respectively, demonstrating reliability and reproducibility.

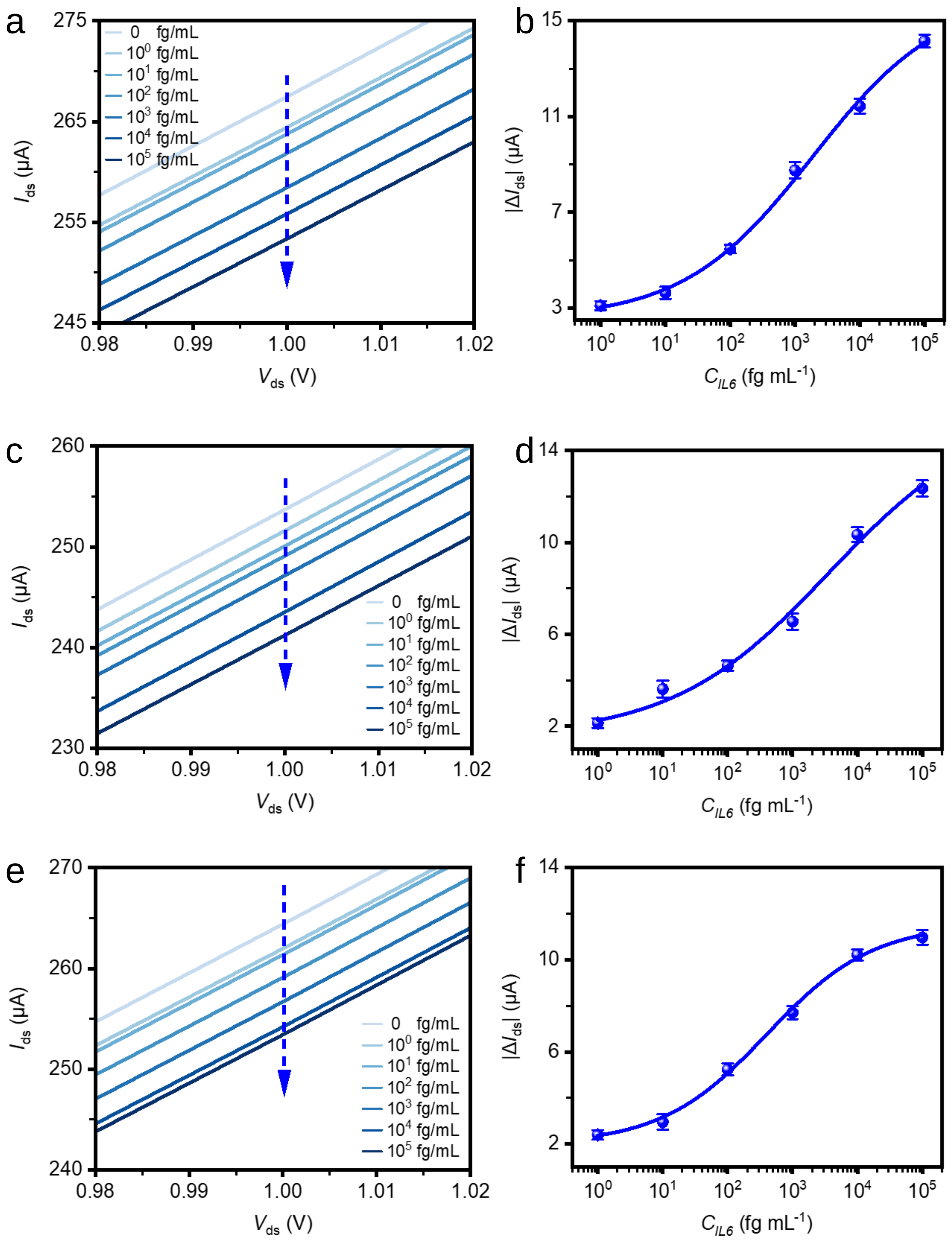

Figure S8. Detection of IL6 using Device-2, 4, 6 (under “-1” mode). The I_{ds}-V_{ds} curves for different IL6 concentrations (at incubation time of 5 min) of (a) Device-2 (shown as Figure 6c in paper), (c) Device-4, and (e) Device-6. The standard curves of (b) Device-2 (shown as Figure 6d in paper), (d) Device-4, and (f) Device-6 with $|\Delta I_{ds}|$ of 14.1 μA, 12.4 μA, and 11.0 μA, respectively, showing reliability and reproducibility.

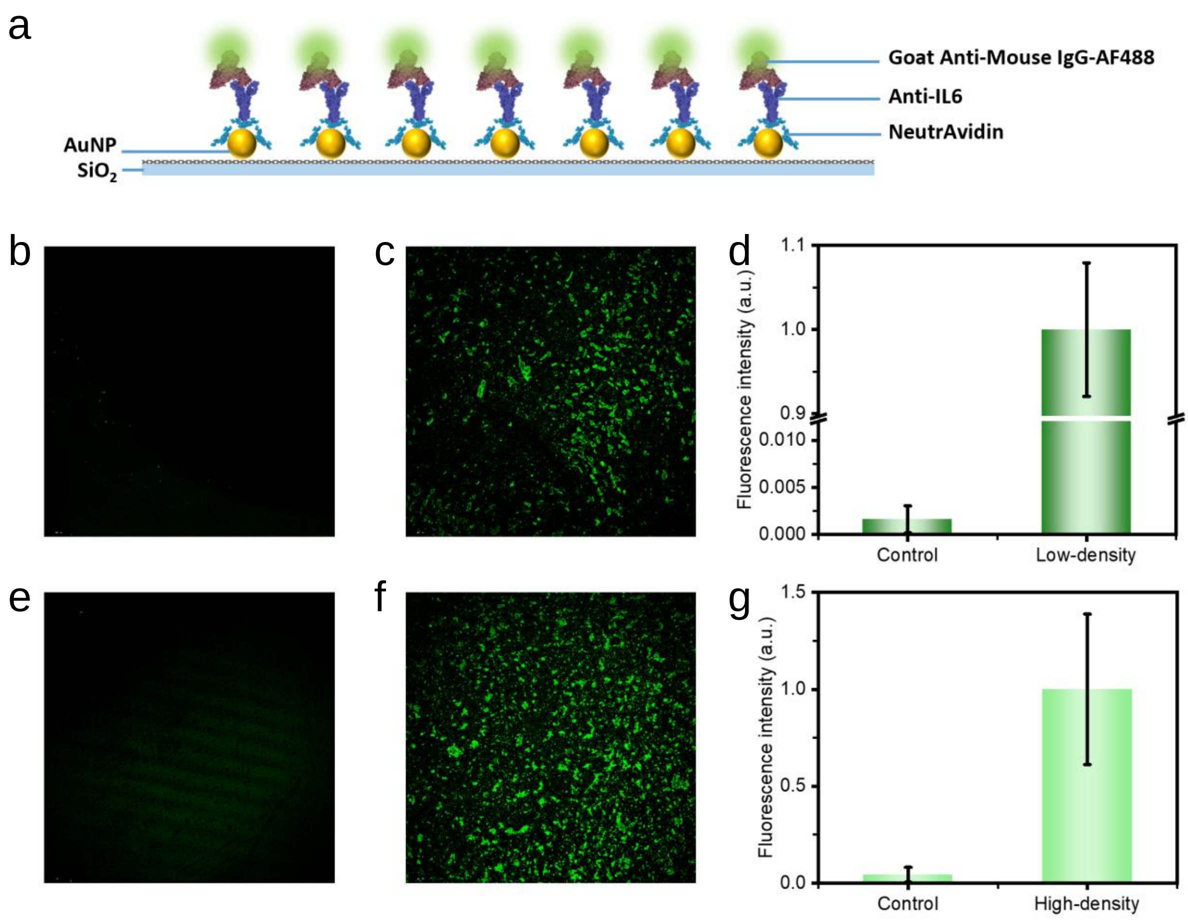

Figure S9. Fluorescent characterization of Mouse biotinylated anti-human IL6 antibody (anti-IL6) on AuNPs filter. (a) Schematic of secondary fluorescent antibody (Goat Anti-Mouse IgG-AF488) binding with anti-IL6. For low-density AuNP filter: (b) control group, (c) fluorescence microscopic image, (d) statistical values of fluorescence intensity in (b) and (c). For high-density AuNP filter: (e) control group, (f) fluorescence microscopic image, (g) statistical values of fluorescence intensity in (e) and (f).
